# Prognostic Factors of Outcome in Methanol Poisoning; an 8-year Retrospective Cross-sectional Study

**Published:** 2020-09-05

**Authors:** Vahid Yousefinejad, Badia Moradi, Anvar Mohammadi Baneh, Farshad Sheikhesmaeili, Asrin Babahajian

**Affiliations:** 1 Liver and Digestive Research Center, Research Institute for Health Development, Kurdistan University of Medical Sciences, Sanandaj, Iran.; 2 Student Research Committee, Kurdistan University of Medical Sciences, Sanandaj, Iran.; 3 Internal Medicine Department, Faculty of Medicine, Kurdistan University of Medical Sciences, Sanandaj, Iran.

**Keywords:** Poisoning, methanol, Prognosis, Outcome Assessment, Health Care

## Abstract

**Introduction:**

Identification of high-risk patients with poor prognosis is essential for quick diagnosis and treatment of methanol poisoning to prevent death and improve the outcome. The aim of this study was to evaluate the clinical and laboratory factors in patients with methanol poisoning to determine the prognosis and outcome.

**Methods:**

In this retrospective cross-sectional study, all patients with methanol poisoning, who had presented to the emergency department of Tohid Hospital, Sanandaj, Iran from 2011 to 2019 (8 years) were enrolled using census method. Multivariate logistic regression analysis was performed to find the independent predictive factors of poor outcome in the mentioned patients.

**Result:**

Methanol poisoning was diagnosed in 52 (11.55%) of the 450 cases admitted to hospital for alcohol intoxication. In multivariate analysis, time interval from methanol intake to hospital admission (OR=1.06; 95% CI= 1.00-1.11; p=0.04), respiratory arrest (OR=25.59; 95% CI= 1.37-478.13; p=0.03), and higher concentration of blood glucose (OR=1.03; 95% CI= 1.00-1.09; p=0.03) had a significant correlation with Poor outcomes.

**Conclusions:**

Based on the findings of this study, delayed admission to hospital, respiratory arrest and *hyperglycemia* were identified as independent risk factors of poor outcome in *methanol poisoning.*

## Introduction

Methanol poisoning due to drinking illicit and homemade alcoholic beverages is a major medical problem worldwide (1-3) and despite advances in diagnosis and treatment, mortality rate in such patients is high (4) . If treatment is delayed or inadequate, mortality rate may reach up to 40%, and even if the patients survive, poisoning may lead to permanent blindness and long-term effects on their central nervous system (5-7). 

The symptoms of methanol poisoning appear 12 to 24 hours after its intake, because its toxic effects are due to toxic metabolites of methanol and not methanol itself. Moreover, in case of delayed hospitalization and treatment, severe metabolic acidosis occurs due to transformation of methanol to toxic metabolites (8). On the other hand, clinical symptoms (*abdominal pain*, shortness of breath/hyperventilation and visual disturbances) may mimic the signs and symptoms of other diseases. In fact, many patients die before reaching the *hospital* and being diagnosed with methanol poisoning (1, 2, 9). 

Under Iranian law, the sale, purchase and intake of alcoholic drinks are illegal and punishable. As a result, people who want to drink alcohol use industrial or homemade alcohol, which is sometimes a mixture of methanol and ethanol (10, 11). On the other hand, some people use alcohol containing methanol due to prohibition of the sale of alcoholic beverages or they are accidentally poisoned by this type of alcohol; however, they do not seek medical treatment due to social shame and fear of legal punishment. 

Methanol poisoning is increasing in recent years due to counterfeit alcohol intake, and fear of legal punishment and delay in the onset of the poisoning symptoms may delay patients' referral to the hospital and thereby increase their mortality rate. Whereas, rapid diagnosis and treatment are necessary to prevent death and to minimize neurological complications. Therefore, researchers around the world are looking for methods to quickly identify high-risk patients with poor prognosis. In previous studies, factors such as delayed hospitalization after alcohol consumption, coma or seizures on admission, severe metabolic acidosis, and inadequate hyperventilation have been identified as indicators of poor prognosis in methanol poisoning (11-13). In Iran, there have been few studies on clinical and epidemiological findings in groups of people who had experienced methanol poisoning, most of which were performed on small groups and paid little attention to para-clinical parameters (10, 11). This study investigated the clinical factors and para-clinical findings to determine the prognosis of methanol poisoning in patients who referred to Tohid Hospital, Sanandaj, Iran, over eight years (2011-2018).

## Methods

***Study design and Setting***

In this retrospective cross-sectional study, patients with methanol intoxication presenting to emergency department of Tohid Hospital, Sanandaj, Iran, from 2011 to 2019 (8 years) were studied. The protocol of this study was approved by the Ethics Committee of Kurdistan University of Medical Sciences (IR.MUK.REC.1397/331) and the principles of confidentiality of information were respected by the researchers according to the Helsinki Declaration.

***Participants***

The medical records of patients presenting with alcohol intoxication were reviewed and those who had been treated with a diagnosis of methanol poisoning (based on clinical symptoms) were enrolled using census method and patients were excluded from the study if they had died before being evaluated.

***Data gathering***

Data collection was done using a checklist containing information regarding demographic data (age, sex); clinical data including concomitant use of opioids or psychoactive drugs, suicide attempt, time interval between methanol intake and hospital admission, level of consciousness, visual disturbances, number of hemodialysis sessions, ventilation and administration of antidote (ethanol) in patients and laboratory findings including pH, PCO_2_, Osmolality Gap, HCO_3_, creatinine (Cr), potassium (K) and blood sugar (BS). Data collection was performed by an internal medicine resident. Data were extracted from medical records of admitted patients. 

***Outcome***

To evaluate outcomes, Patients were divided into two groups: poor outcome (survivors with sequelae and those who died) and good outcome (survivors without sequelae).

***Statistical Analysis***

SPSS software version 20 was used for data management and analysis. Quantitative variables were reported as mean ± standard deviation (SD), and median and interquartile range (IQR) for data with normal and abnormal distribution, respectively. Qualitative variables were presented as frequency (percentage). In addition, Mann-Whitney U test, t-test, and Chi-square test were used to evaluate data with abnormal distribution, assess data with normal distribution, and compare categorical variables, respectively. A multivariate logistic regression model was applied to find the factors related to the outcome. P-values less than 0.05 were considered statistically significant. 

## Results

***Demographic and clinical characteristics of patients***

Of the 450 cases admitted to the Emergency Department of Tohid Hospital in Sanandaj, Iran, due to alcohol intoxication during the 8-year period, 52 (11.55%) were diagnosed with methanol intoxication. All of the patients were male; with the mean age of 32.78± 12.30 years (range 14-60 years).

According to the medical records, 4 (7.7%) patients had attempted suicide by using alcohol (containing methanol), one of which died. Concomitant use of opioids or psychoactive drugs was reported in 9 (17.3%) cases.

The median time interval between methanol intake and hospital admission was 24 hours (range: 4 to 48 hours). On admission to the emergency department, 32 (61.5%) had visual disturbances. The patients’ consciousness state was evaluated and 32 (61.5%) were awake, 17 (32.7%) were comatose (GCS less than 8), and 3 (5.8%) were awake-coma (lost their consciousness* after* *hospitalization)*. 10 (19.2%) patients needed ventilation due to respiratory arrest.

Hemodialysis was performed in all patients. The median time interval between hospital admission and beginning of hemodialysis was 2 hours, with the range of 1-3 hours. 14 patients (26.9%) underwent hemodialysis more than once.

13 (25%) patients were treated with ethanol as an antidote. A total of 12 (23.1%) patients were admitted to intensive care unit (ICU).

Finally, death occurred in 8 (15.4%) patients, 4 (7.7%) survivors had ocular and cerebral sequelae, and other patients (76.9%) were discharged with complete recovery.

***Factors associated with poor outcomes***

Details of demographic and clinical factors associated with poor outcome are shown in Table1. The median time interval between methanol intake and hospital admission was higher in patients with poor outcome (48 hours, IQR: 26-72 hours) compared to those who recovered (18 hours, IQR; 3-48) (p< 0.01). Respiratory arrest rate on admission was also significantly higher in poor outcome group; only 3 (30%) patients who had respiratory arrest on arrival survived without sequelae (p < 0.001).

Table 2 shows the laboratory findings. The concentrations of glucose (p<0.001), creatinine (p<0.01) and *potassium* *(p=0.01)* were higher in the poor outcome group. In contrast, pH (p= 0.09), pCO_2_ (p= 0.50), HCO_3_ (p= 0.06), and osmolality gap (p= 0.30) did not have a significant association with poor outcome.

Additionally, in multiple logistic regression analysis, increased time interval between intake and admission (OR = 1.06; 95% CI: 1.00 to 1.11; p = 0.04), respiratory arrest (OR = 25.59; 95% CI: 1.37 to 478.13; p = 0.03), and higher *blood sugar level* (OR = 1.03; 95% CI: 1.00 to 1.09; p = 0.03) remained independently associated with poor outcome (Table 3).

## Discussion

In this study, clinical and para-clinical factors affecting the prognosis of methanol poisoning were investigated. The main findings indicated that methanol poisoning was diagnosed in only 11.55% of those admitted to Tohid Hospital in Sanandaj, Iran, due to alcohol poisoning over 8 years. Delay in transfer to hospital, respiratory arrest on admission, and hyperglycemia were associated with poor prognosis (death and Sequelae).

In previous studies, respiratory arrest (1, 2, 11, 14) as well as more than 24 hours passing from methanol intake at the time of hospital admission (11) had been identified as factors predicting poor prognosis for methanol poisoning, which is consistent with our study.

Moreover, patients were asymptomatic 12 to 24 hours after methanol intake and this period was defined as the latent period. This latent period was most likely related to the period when methanol was metabolized to formaldehyde and formic acid (15). In an eight-year study, Yaycı et al. reported delayed hospitalization to be the cause of methanol poisoning deaths in 77.5% of patients (16) . Najari et al., in their study, pointed to the causes of delay in initiation of treatment, which leads to long-term complications and mortality in patients, and cited difficult diagnosis of intoxication due to non-specific signs and symptoms, delay in patient’s referral, and the impossibility of obtaining a proper history of the patient (17). In the present study, the median time interval between methanol intake and hospital admission in patients with poor prognosis (morbidity and mortality) was greater than those who completely recovered. 

Our study also showed that elevated serum levels of BS was associated with poor prognosis. In a retrospective study, Sanaei-Zadeh et al. examined 95 people who had been treated for methanol poisoning at Loghman and Hazrat Rasoul Hospitals between 2003 and 2010 in Tehran, Iran. They reported that blood serum glucose levels in those who died (219±99 mg/dl) was significantly higher than those who survived (140±55 mg/dl) (p <0.001) and reported that among the factors studied, serum glucose levels above 140 were a risk factor in predicting mortality in these patients (14). 

The mechanism of hyperglycemia in methanol poisoning is unclear. Methanol poisoning has been reported to be associated with acute pancreatitis (18, 19), which can play a role in hyperglycemia. Another mechanism that may lead to an increase in blood glucose levels in methanol poisoning is stress-induced hyperglycemia, which is commonly seen in critically ill patients. In other words, increased levels of acute stress hormones may play a role in development of methanol poisoning. Therefore, insulin therapy and blood sugar control may be applied in the management of methanol poisoning (20, 21) .

In the present study, all the subjects were male, which indicates that methanol abuse in our country occurs predominantly in men. In the study of Hassanian et al., 23 of the 25 cases of methanol poisoning were male (11) . Other studies have shown that men are more likely to be poisoned with methanol and die from it. In these studies, the higher prevalence of alcohol consumption in men compared to women was reported as the reason for the higher prevalence of methanol poisoning in men (22, 23).

In addition, ocular symptoms were reported as one of the major symptoms of methanol poisoning associated with retinal toxicity, which ranged from blurred vision, changes in visual field, photophobia, difficulty in adjusting light and double vision to complete blindness and Nystagmus, which was uncommon. In this study, 61% of patients had visual impairment on admission and findings from other studies also reported visual impairments in 29–77% of all patients (1, 24, 25).

**Table1:** Demographic and clinical characteristics associated with poor outcome in patients with methanol poisoning

**p-value**	**Outcome**		**Variables**
	**Poor n=12**	**Good n=40**	
			**Age (year)**
0.14	37.5 (25.3 – 49.3)	29.5 (23.0 – 38.8)	Median (IQR)
			**Location**
	12 (100.0)	35 (87.5)	Urban
0.58	0 (0.0)	5(12.5)	Rural
		**Intake to admission ** **(** **hour** **)**	
< 0.01	48.0 (26.0 – 72.0)	18.0 (3.0 – 48.0)	Median (IQR)
		**Admission to hemodialysis ** **(** **hour** **)**	
0.98	2.0 (0.6 – 3.8)	2.0 (1.0 – 3.0)	Median (IQR)
			**Suicide**
	1 (8.3)	3 (7.5)	Yes
> 0.99^¥^	11 (91.7)	37 (92.5)	No
		**Concomitant use of opioids or psychoactive drugs**	
	2 (16.7)	7 (17.5)	Yes
> 0.99^¥^	10(83.3)	33(82.5)	No
			**Coma on admission**
	6 (50.0)	11 (27.5)	Yes
0.17^¥^	6 (50.0)	29 (72.5)	No
			**Visual Symptoms on** ** admission**
	8 (66.7)	24 (60.0)	Yes
0.74^¥^	4 (33.3)	16 (40.0)	No
			***Frequency of hemodialysis***
	6 (50.0)	32 (80.0)	once
0.06^¥^	6 (50.0)	8 (20.0)	More than once
			**Antidote (ethanol)**
	4 (33.3)	9 (22.5)	Yes
0.46^¥^	8 (66.7)	31 (77.5)	No
			**Respiratory arrest on admission**
	7 (58.3)	3 (7.5)	Yes
< 0.001^¥^	5 (41.7)	37 (92.5)	No

Continuous variables presented as Median (interquartile range; IQR) and Categorical variables are presented as frequency (%).

Mann-Whitney U test was used for analysis.

¥ Fisher’s exact test was used for analysis.

**Table2:** Laboratory factors associated with poor outcome in patients with methanol poisoning

**P value**	**Outcome**		**Variables**
	**Poor** ** (n** **=12)**	**Good** ** (n** **=40)**	
0.09	7.10 (6.90 – 7.30)	7.28 (7.11 – 7.30)	pH
0.06	7.30 (5.92 – 16.42)	13.00 (7.90 – 19.55)	HCO_3_ (mmol/L)
0.50	28.01 ± (8.08)	30.43 ± 10.66	pCO_2_ (kPa)
0.30	299.14 ± 14.50	295.41± 8.94	Osmolality Gap
< 0.001	163.0 (156.0 – 323.0)	116.0 (104.0 – 136.0)	BS (mg/dL)
0.01	4.83 ± 0.89	4.07 ± 0.82	K (mmol/L)
< 0.01	1.41 ± 0.49	1.08 ± 0.32	Cr (mol/L)

* Continuous variables presented as Mean ± standard deviation (SD) for normally distributed variables or median (interquartile range; IQR) for data that are not normally distributed. BS: blood sugar; K: Potassium; Cr: Creatinine.

one-way Anova was used for analysis.

Kruskal-Wallis was used for analysis.

**Table 3:** The results of multivariate logistic regression analysis on the factors associated with a poor outcome in patients with methanol poisoning

**P-value**	**95% CI**	**OR**	**Variables**
0.03	1.37 - 478.13	25.59	Respiratory arrest
0.04	1.00 - 1.11	1.06	Intake to hospital admission
0.03	1.00 - 1.09	1.03	High blood glucose level

OR: Odds Ratio; CI: confidence interval.

## Limitation

Limitations of this study included small sample size, the retrospective nature of the study, and the short-term follow-up. In addition, due to the lack of measurement of serum methanol levels in patients, diagnosis was based on clinical findings in patients.

## Conclusions:

Based on the findings of this study, delayed admission to hospital, respiratory arrest, and *hyperglycemia* were identified as independent risk factors of poor outcome in *methanol poisoning.*
